# Seroprevalence of sheeppox and goatpox virus in Asia and African continent: A systematic review and meta-analysis (Scientometrics)

**DOI:** 10.14202/vetworld.2022.455-464

**Published:** 2022-02-25

**Authors:** Kuralayanapalya Puttahonnappa Suresh, Anenahalli Panduranga Bhavya, Chandan Shivamallu, Raghu Ram Achar, Ekaterina Silina, Victor Stupin, Shiva Prasad Kollur, Bibek Ranjan Shome, Sharanagouda S. Patil

**Affiliations:** 1 ICAR-National Institute of Veterinary Epidemiology and Disease Informatics (NIVEDI), Yelahanka, Bengaluru, Karnataka, India; 2 Department of Biotechnology and Bioinformatics, School of Life Sciences, JSS Academy of Higher Education and Research, Mysore, Karnataka, India; 3 Division of Biochemistry, School of Life Sciences, JSS Academy of Higher Education and Research, Mysuru, India; 4 Department of Human Pathology, I.M. Sechenov First Moscow State Medical University (Sechenov University), Moscow, Russia; 5 Department of Hospital Surgery, N.I. Pirogov Russian National Research Medical University (RNRMU), Moscow, Russia; 6 Department of Sciences, Amrita School of Arts and Sciences, Amrita Vishwa Vidyapeetham, Mysuru, India

**Keywords:** Asia, Africa, capripoxvirus, meta-analysis, seroprevalence, subgroup analysis

## Abstract

**Background and Aim:**

Two endemic capripox infectious diseases, sheeppox (SP) and goatpox (GP) are common in Asia, Africa, and the Middle East. Sheep and goats, in general, are considered current assets of small and marginal farmers and have significant economic value in terms of meat, wool, and skin/hide production. Sheep and goat populations in India total 148.88 million and 74.26 million, respectively. Capripox caused US$ 2.3 million (Indian Rupee [INR] 105 million) in economic damages in Maharashtra (India) alone, and it took over 6 years for a flock to recover from the outbreak. The projected yearly loss at the national level is US$ 27.47 million (INR 1250 million). As a result, Capripox diseases put small and marginal farmers under much financial strain. The present study estimates the seroprevalence of SP and GP diseases in the Asian and African continents using systematic review and meta-analysis. The results of the study will help researchers and policymakers to understand the spatial and temporal distribution of the disease and its burden. In addition, the results are also helpful to design and implement location-specific prevention and eradication measures against these diseases.

**Materials and Methods:**

Preferred Reporting Items for Systematic Reviews and Meta-Analysis guidelines of Cochran collaborations were used for systematic review and subsequently meta-analysis were used. The literature was collected from various databases. Initial search string resulted in more than nine thousand articles for the period 2000 to 2020 using the different combinations of keywords and Boolean operators (or not) asterisk* and quotation marks. Out of 9398 papers, 80 studies were chosen for complete test reviews and quality bias evaluation using the inclusion and exclusion criteria. Finally, 21 articles were used for the meta-analysis. The statistical study employed fixed effects and random effects models using R.

**Results:**

Seroprevalence of SP and GP was calculated using studies with a cumulative sample size of 4352, out of which sheep and goats’ samples together contribute 48%, followed by sheep (32%) and goat (21%). The result of the meta-regression revealed that detection techniques had a significant impact on the overall effect size at 5% level (Qm=14.12). Subgroup analysis of polymerase chain reaction (PCR) test with samples was further grouped into two categories based on the median, and it revealed that 62% of samples used PCR as a detecting test followed by group-II.

**Conclusion:**

From the study, it is concluded that SP and GP diseases are highly prevalent; hence, effective vaccines, proper education to farmers through extension activity, and transboundary disease movement restriction are necessary for the control and eradication of the disease.

## Introduction

*Capripoxvirus* (Poxviride) is a notifiable disease [1] due to its impact on small ruminant productivity. The family Poxviridae includes sheeppox virus (SPPV), goatpox virus (GTPV), and Lumpy skin diseases virus, and all these viruses are closely related serologically and have indistinguishable share over 96% sequence homology among themselves [2]. SPPV and GTPV are -borne diseases that primarily affect sheep and goats, respectively [3].

In general, both SPPV and GTPV are host-specific, but some strains are ineffective to heterologous hosts also. However, recently molecular basis of host specificity study shows some genes are associated with conferring host preference [4,5].

The virus spreads through aerosols generated from infected animals or through direct abraded skin/mucosal contact or indirectly through mechanical transmission by vectors [1,6]. There is no evidence that human infection with capripoxvirus and capripoxvirus is not pathogenic to humans [2,7]. The clinical signs in SPPV and GTPV are characterized by mild to severe fever, erythematous macules, vesicles, papules, pustules, and scabs on the skin. The lesions may also develop on the mucous membrane and internal organs, causing respiratory signs, diarrhea, depression, emaciation, and abortion [2,5]. The mortality rate is highly variable (ranging from 5 to 10% in local breeds in endemic regions, to 100% of exotic sheep and goat species in endemic regions [8], SPPV and GTPV outbreaks have become a major concern in Northern and Central Africa, the Middle East, and most of the Asian continent [2,3]. In India, goatpox (GP) outbreak was first reported in the year 1936, and sheeppox (SP) was first reported in Bombay (1931-1932) and Mysore [9]. Since then, frequent outbreaks have been reported from several states, causing significant economic losses [8,10-12]. A recent outbreak was reported in Himalayan gorals during April-May 2018 in the Tawang district of Arunachal Pradesh [13]. In the case of the African continent, Ethiopia is believed to have the largest livestock population with more than 49 million sheep and goat population [14]; a total of 663 SP and GP disease outbreaks were reported in all major parts of Eastern Amhara Region, Ethiopia between 2013 and 2019. From these outbreaks, 57,638 sheep and goats contracted the disease. Out of the 57,638 sick sheep and goats, 6401 animals died [15]. A study was conducted on herds and flocks affected between August 2017 and January 2018 in Bauchi, Nigeria, revealed that incidence risk and fatality rate were 53 and 34% in sheep; 50 and 33% in goats, respectively Limon *et al*. [16]. Seroprevalence of SP and GP in three states of Northern Nigeria (Bauchi, Kaduna, and Plateau) was 2% at a 95% confidence interval (CI) [17]. Recently outbreaks have been reported in Kazakhstan, Mongolia, Azerbaijan, Turkey, Greece, and Bulgaria of the Asian continent [18].

Enzootic capripox is responsible for direct and indirect economic loses in sheep and goats and it causes productivity losses in endemic areas, including reduced milk yield, reduced weight gain, increased abortion rates, damage to wool and hide, and increased susceptibility to pneumonia and flystrike along with mortality [19]. In addition to the above, it also reduced the free trade of animals and animal products from endemic areas to other places [12]. The economic losses due to capripox in Maharashtra (India) state alone was US$ 2.3million (Indian Rupee [INR] 105 million) and it took nearly 6 years for a flock to recover from capripox outbreak [20]; estimated annual loss at a national level is US$ 27.47 million (INR 1250 million) [20]. India has 148.88 million and 74.26 million populations of sheep and goat, respectively [20]. In the case of Ethiopia, nearly 5-7 million sheep and goat die annually and the estimated economic loss to meet industry is US$ 400 million annually [21].

Hence, considering the sheep and GP’s economic importance is crucial for estimating the disease burden in the region. Keeping the above factors in mind, the present epidemiological study estimates the prevalence of SP and GP diseases in Asian and African continents based on systematic review and meta-analysis. The result could be an input for the researcher and policymakers about the disease (SGP) burden, thereby supporting the process of identification of priorities in veterinary healthcare, prevention, and policy.

## Materials and Methods

### Ethical approval

Ethical approval was not necessary as the study materials were collected through the public literature database.

### Study period and location

The present study included the literature which were collected from various databases for the period 2000 to 2020 in Asia and Africa.

### Literature search strategy

Preferred Reporting Items for Systematic Reviews and Meta-Analysis guidelines of Cochran collaborations were used for systematic review and subsequently meta-analysis [22-24]. A literature survey was performed systematically to collect relevant literature on the prevalence of sheep (SPPV) pox and GTPV in the Asian and African continent. The information was collected from the various databases, including PubMed, Google Scholars, Science Direct, Springer’s, Biomed Central, Consortium of e-Resources in Agriculture, research proceedings/compendium of conferences, seminars, symposia, Krishikosh, and other published sources. Initial search string resulted in more than nine thousand articles for the period 2000 to 2020 using the different combinations of keywords [SP, GP, Capripoxvirus, prevalence, seroprevalence, ovine, caprine, small ruminant, epidemiology, and domestic ruminant] along with Boolean operators [or and not] asterisk* and quotation marks [“ “] were used to Peer-reviewed publication with the English language were retained. Zotero 5.0 developed by George Mason University, Virginia, USA and Rayyan developed by Qatar Computing Research Institute, Qatar (the Systematic Reviews web app) were used for systematic reviews and compilations [25].

### Study selection and data extraction

**Figure-1:**
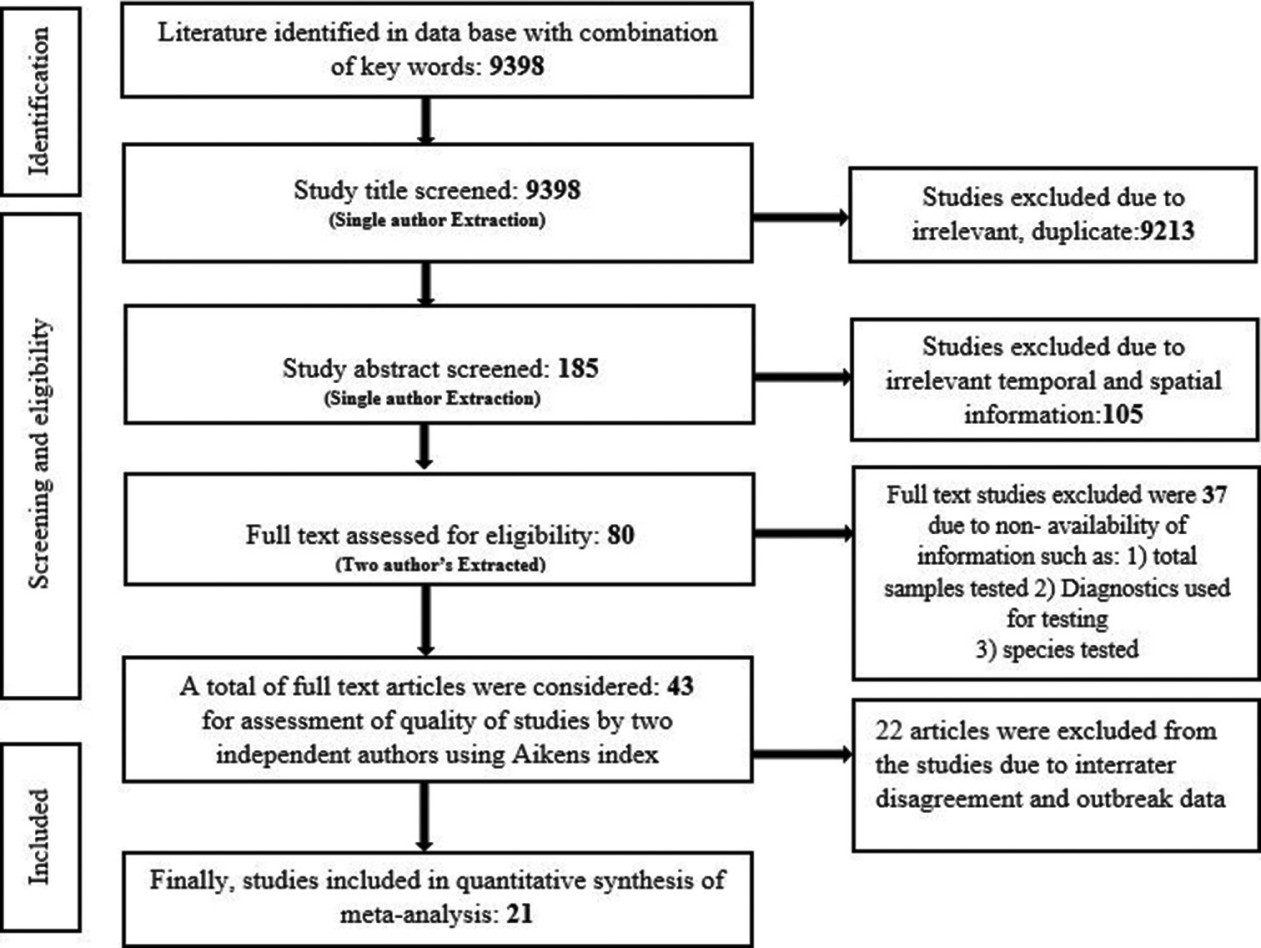
Schematic diagram of selection of articles used for the systematic review of this study.

The schematic representation of the systematic review on seroprevalence of SP and GP in Asia and the African continent is depicted in Figure-1. Out of the 9398 studies compiled during the literature search; During the preliminary screening phase, studies were excluded based on irrelevance, duplicates, lack of temporal and spatial information, etc. Accordingly, 80 studies were selected for full-text reviews and subjected to quality of bias assessment. Finally, 21 articles were used for the meta-analysis [5,21,26-44]. The determinants such as author, publication year, research conducted year, region, species (sheep and goat), number of the sample tested, number of positive samples, and tests used for the analysis were extracted from the selected articles.

### Quality assessment of studies

Quality assessment of the studies was done by two investigators independently. Investigator used the seven questions with 5 points Likert scale to judge the quality of each research paper. The maximum score of five indicates a very likely and 1 very unlikely article. The scores of the investigators were further used to calculate the coefficient of the validity with Aiken value [44-46].

V=∑S/[[n*[c-1]]]

Where,

Aiken V=Validity index

S=Scores assigned by each rater minus the lowest score in the used

S=r-lo

r=rater category selection score

lo=lowest scores in the scoring category

c=Maximum score in the grading scale

n=number of rater

V value ranges from 0 to 1, where 1 indicate rater gives 100% consent to included studies concerning structured question included.

### Statistical modeling and analysis

#### Meta-analysis

Meta-analysis is a statistical research process used to assimilate various studies to calculate an overall summary estimate of the study using R open-source scripting software written by R core team (Comprehensive R Archive Network) version 3.2.5 and the R package used was “meta” as reported earlier [47]. The graphical representation of effect size was done through forest plot or CI plot. In a meta-analysis, predominantly fixed effect and Random effect models are used based on the variation in the studies and inconsistency (I^2^) values. The random effect model will be used when the heterogeneity among the studies is found statistically significant in combination with inconsistency indices.

#### Quantifying heterogeneity and Inconsistency

The degree of heterogeneity in a meta-analysis decides the effort in reaching general interpretations. This degree might be estimated by assessing the variance between the different studies [48,49]. Indices H and I^2^ values are usually calculated to summarize the impact of heterogeneity among included studies [50]. Inconsistency (I^2^) measures the degree of inconsistency ranging from 0 to 100%. Where I^2^ is preferable to test for heterogeneity in judging consistency of evidence and selection of either fixed-effect or random-effect model. If I^2^ <50% it signifies least heterogeneity, I^2^ >50% least-moderate heterogeneity, and I^2^ >95% indicates high heterogeneity [25,46] in the analysis.

#### Testing of heterogeneity

It is important to consider the inconsistency among the studies to calculate heterogeneity. If CI for the results of individual studies (generally depicted graphically using horizontal lines) have poor overlap, this generally indicates the presence of statistical heterogeneity [46]. This can be calculated using Cochran’s Q statistic, Tau square, H value, and p values obtained, and results are given in the last line of the forest plot [25,51]. The calculated Chi-squared (χ^2^, or Chi-square) test is included in the forest plots in Cochrane reviews [52,53] helps to assess whether observed differences in results are compatible with chance alone or not and p<0.05 is considered to have a presence of heterogeneity.

#### Meta-regression

Meta-regression is conducted to analyze the influence of included studies with respect to estimates and variation in the studies [54]. Large-scale investigations have more impact as they are weighted by the exactness of their impact estimate [47]. It needs to consider the leftover heterogeneity among intercession impacts of those which are not exhibited by the variable [55].

To predict the effect of a hypothesized moderator, a weighted linear regression model was applied in which the effect sizes (Samples) were regressed onto the moderator [47,50]. The moderators included in univariate meta-regression were the serological test, geographic region, Species, year, and sample size. The variables with p<0.05 in univariate meta-regression were used in multivariable meta-regression, and only factors significant at p≤0.05 were retained in the final model. Meta-regression reduces the number of tests and estimations; hence, the power of analysis is greater, and the probability of false-positives findings is reduced [54].

#### Subgroup analysis

**Figure-2:**
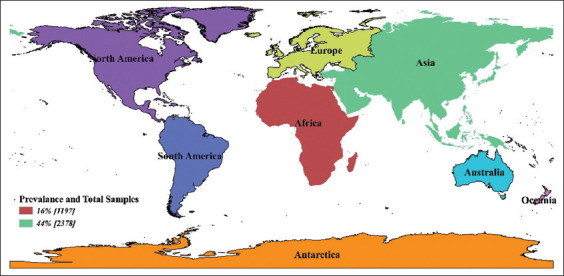
Prevalence of sheeppox and goatpox in Asian and African continent [Source: Map was prepared by the authors].

Subgroup analysis is the procedure where all collected data incorporated in the meta-analysis are divided into different groups as driven by meta-regression [25]. It helps control the heterogeneity in the studies. Variables required for subgroup analysis are selected based on R^2^ values and the significance of meta-regression. In our study, subgroup analysis was done to assess the heterogeneity in the region (Asia and Africa) (Figure-2), sample with different tests, and polymerase chain reaction (PCR) with samples [49,50].

#### Sensitivity analysis

Sensitivity analysis determines how different values of an independent variable affect a particular dependent variable under a given set of assumptions. In the present study, sensitivity analysis was performed to identify the studies which contribute to overall heterogeneity and measure the robustness of meta-analysis findings [49].

#### Publication bias

**Figure-3:**
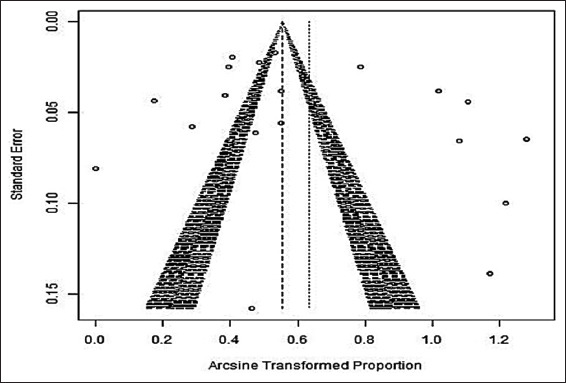
Publication bias among studies is shown in funnel plot showing asymmetry and heterogeneity.

Publication bias is a critical problem in systematic review and meta-analysis, which can affect the validity and generalization of conclusions [52]. The literature has high positive tested samples along with new confirmatory tests have a high chance of publishing this implied bias toward the result. In this study funnel, plot-based methods include a visual examination of a funnel plot, regression, and rank test were used to assess publication bias. A funnel plot plotted with arcsine transformation proportion in the X-axis and standard error in Y-axis. The arcsine-based transformation has the advantage of stabilizing variance [52], which is likely the main reason for our study. If the publication bias is nil, high-accuracy investigations lie along the line of normal. In contrast, low-accuracy investigations scatter equitably on both margins of the normal line, making a funnel-shaped distribution [50]. The dispersion of publications in the funnel formation directs to publication bias. In Figure-3, most of the studies were scattered and a few of the studies fall into the funnel, indicating the publication bias. The presence of asymmetry in the funnel plot was tested using Beggs rank correlation test (Kendall’s tau=0.15 with p=0.3536) and Eggers regression test (z=8.51, p<0.010). To deal with the presence of publication bias, we have employed the meta-regression with sample size as the risk of bias factor, proving the non-significance (p>0.05) nullifying the effect of publication bias in the study.

## Results

### Bias assessment

The seroprevalence of SP and GP was calculated using a total sample size of 4352, out of which sheep and goats’ samples together contribute 2084, followed by sheep (1367) and goat (901). The quality of the studies was assessed and the same is presented in Table-1, which indicates the score given by the two independent authors with respect to seven items using the Likert scale. Based on the ratings calculated, the Aiken V value for all the studies is more than 0.7, and it indicates that the study quality is acceptable.

**Table 1:** Studies included in the meta-analysis with their quality assessment scores.

Study	Region	Country	Study’s target population is a representative of the national population with respect to relevant variables?	How were the samples selected, randomly or census undertaken?	Was the probability of bias was minimal among the studies?	Was the data collected directly from the subjects?	Was an acceptable case definition used in the study?	Was the measured parameter valid and reliable?	Mode of sample collection was same for all the studies?	Summary on the overall risk of study bias	Aiken V Index
							
			Average score*	Average score*	Average score*	Average score*	Average score*	Average score*	Average score*	Average score*	
Boshra *et al*. [5]	Asia	Saudi Arabia	4.00	4.50	4.50	4.50	4.50	5.00	4.50	4.50	0.88
Fentie *et al*. [21]	Africa	Ethiopia	4.50	5.00	5.00	5.00	5.00	4.50	5.00	4.86	0.96
Hopker *et al*. [27]	Asia	India	4.50	5.00	5.00	5.00	5.00	4.50	5.00	4.86	0.96
Hota *et al*. [26]	Asia	India	5.00	5.00	5.00	5.00	5.00	4.50	5.00	4.93	0.98
Enan *et al*. [43]	Africa	Sudan	5.00	4.50	4.50	4.50	4.50	5.00	5.00	4.71	0.93
Kali *et al*. [34]	Africa	Algeria	5.00	5.00	5.00	5.00	5.00	5.00	4.00	4.86	0.96
Kardjadj *et al*. [37]	Africa	Algeria	4.50	5.00	4.50	5.00	5.00	5.00	4.50	4.79	0.95
Mansour *et al*. [36]	Africa	Sudan	5.00	4.50	4.50	4.50	4.50	5.00	4.50	4.64	0.91
Masoud *et al*. [42]	Asia	Pakistan	5.00	4.50	4.50	5.00	4.00	4.00	4.50	4.50	0.88
Pham *et al*. [40]	Asia	North Vietnam	4.50	4.00	4.50	5.00	4.00	4.50	4.50	4.43	0.86
Santhamani *et al*. [41]	Asia	India	4.00	4.00	5.00	5.00	5.00	4.50	4.00	4.50	0.88
Soundararajan *et al*. [39]	Asia	India	4.00	4.00	5.00	4.50	5.00	4.50	5.00	4.57	0.89
Ramachandran *et al*. [30]	Asia	India	4.00	5.00	5.00	5.00	5.00	5.00	5.00	4.86	0.96
Kadam [33]	Asia	India	4.00	5.00	4.50	4.50	5.00	5.00	5.00	4.71	0.93
Pothiappan *et al*. [38]	Asia	India	4.50	4.50	5.00	5.00	4.50	5.00	5.00	4.79	0.95
Gnanavel [31]	Asia	India	5.00	4.50	5.00	4.50	4.50	4.50	5.00	4.71	0.93
Chetan [29]	Asia	India	5.00	5.00	4.50	5.00	4.50	5.00	4.50	4.79	0.95
Ijaz *et al*. [32]	Asia	Pakistan	4.50	5.00	5.00	5.00	5.00	5.00	4.50	4.86	0.96
Bolajoka *et al*. [28]	Africa	north central Nigeria	4.50	5.00	4.50	4.50	5.00	4.50	5.00	4.71	0.93
Malmarugan *et al*. [35]	Asia	India	5.00	4.50	4.50	4.50	5.00	5.00	5.00	4.79	0.95

* Average score of two independent authors and Aiken value of 21 articles included in the meta-analysis

### Meta-regression to identify the factors affecting the heterogeneity

Univariate meta-regression was used to identify potential covariates that likely affect the magnitude and direction of the overall estimate of heterogeneity. The result of the meta-regression (Table-2) revealed that detection techniques had a significant impact on the overall heterogeneity at 5% level (Qm=14.12) variables such as region, species, sample size, and year were not statistically significant. The estimated results revealed that the subgroup analysis and sensitivity analysis are required for further fine-tuning of prevalence rates of sheep and GTPV.

**Table 2:** The univariate meta-regression analysis of sheep pox and goat pox virus.

Predictors	Estimate	SE	z-value	τ^2^	I² (%)	H²	R² (%)	Qm	p-value
Region	0.40	0.14	2.82	0.11	99.15	117.91	6.76	2.67	0.101
Test	0.44	0.17	2.49	0.09	98.91	91.57	25.02	13.27	0.038*
Species	0.67	0.12	5.50	0.11	99.06	106.68	8.14	3.96	0.137
Sample Size	0.00	0.00	-1.66	0.11	99.14	116.76	7.76	2.77	0.095
Year	-0.01	0.02	-0.66	0.12	99.28	138.47	0.00	0.43	0.510

* Indicate the 5% level of significance

### Sub-group and sensitivity analysis

**Figure-4:**
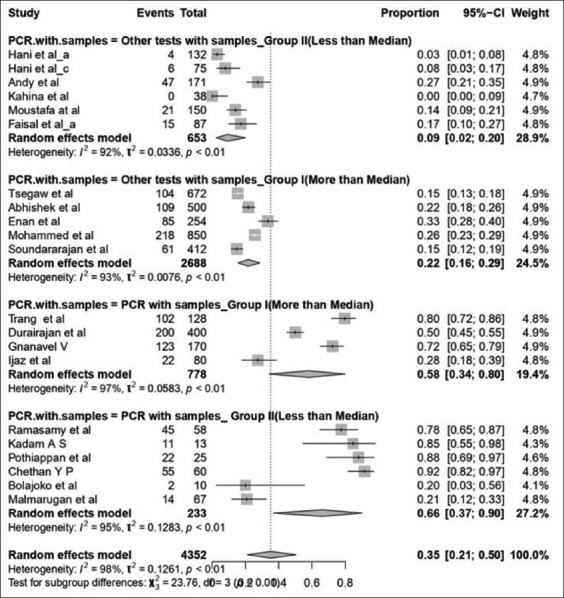
Forest plot for prevalence of sheeppox and goatpox in Asia and African continent based on test with samples.

Subgroup analysis was performed for the covariates like PCR test with a level of sample size, other tests with sample size and region as they could affect the heterogeneity (Table-3). Subgroup analysis of PCR test with levels of samples size was further grouped into two categories based on the median and it revealed that 62% of the sample size used PCR as detecting test in the Group-I category with 95% CI (0.16; 0.296), I^2^=95% and τ^2^=0.030, followed by Group-II 45% prevalence (95% CI: 0.29; 0.66). In the case of the region, studies showed that the prevalence (Figures-2 and 4) of SP and GP for the study period in Asia was 44% (95% CI, 0.26; 0.63 with I^2^=98% and τ^2^=0.134), followed by Africa with 16% prevalence.

**Table 3:** Prevalence of sheep pox and goat pox were stratified according to diagnostic tests with samples for sub-group analysis.

Particulars	Prevalence % (95% CI)	I^2^(%)	τ^2^	Model
PCR with samples				
Group I (More than median)	0.62 (0.16:0.29)	95	0.030	Random Effect Model
Group II (Less than median)	0.47 (0.29:0.66)	88	0.047	Random Effect Model
Other tests with samples				
Group I (More than median)	0.22 (0.16:0.29)	93	0.007	Random Effect Model
Group II (Less than median)	0.09 (0.02:0.20)	92	0.033	Random Effect Model
Overall effect	0.31 (0.20:0.43)	97	0.081	Random Effect Model
Region with samples				
Asia	0.44 (0.26:0.63)	98	0.134	Random Effect Model
Africa	0.16 (0.05:0.30)	94	0.040	Random Effect Model

Other tests include ELISA, Hemagglutination, Radial hemolysis, VNT, clinical examination. PCR=Polymerase chain reaction

## Discussion

**Figure-5:**
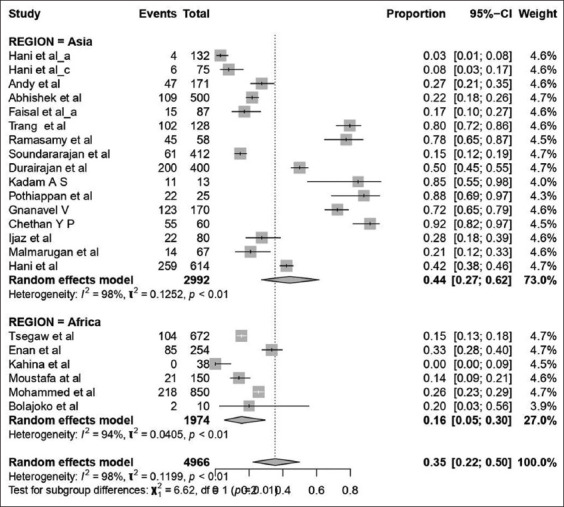
Forest plot for the prevalence of sheeppox and goatpox in Asia and African continent based on region.

Contagious viral infections of SP and GP diseases have been reported in different parts of the world, including the Indian subcontinent and Africa [2,55]. It is more common in the arid and semi-arid zone of East Africa and the Horn of Africa, and also it is endemic in Iran, Iraq, Turkey, Egypt, Sudan, Syria, Southeast Russia, Mongolia, India, Pakistan, Afghanistan, Nepal, Vietnam, Chinese Taipei, and China [5,55,56] This is in accordance with our study of seroprevalence of SP and GP, which were 44 and 16% in Asia and the African continent, respectively (Figure-5).

Commonly, tests such as immunofluorescence, immunoprecipitation, virus neutralization, PCR, and ELISA are employed to detect viral agent/antibodies in disease-endemic areas [57]. However, these tests are insufficient for the detection, sequencing, and differentiation of SP and GTPV. Hence, it is vital to develop and validate the latest PCR assays [58-60] real-time PCR, the loop-mediated isothermal amplification of DNA, nanotechnology-based fluoroimmuno assays [40], ELISAs based on inactivated SPPV antigen [61], recombinant protein and monoclonal antibody-based ELISAs to enable rapid capripoxvirus diagnosis and surveillance in Asian, the African continent. The present study also revealed that PCR tests have significance with the level of sample size compared to other confirmatory tests.

Hence, the present study results are assumed to be significant for the following context;

1. Asia and Africa together contribute more than 75% of the world’s sheep and goat population [62]. Asia alone contributes 43.6 and 55.4% of the world’s sheep and goat population, respectively, which is followed by Africa with 30.0 and 38.7% of sheep and goat population, respectively [63]. The statistics showed the importance of sheep and goats with respect to nutritional (meat and milk) and economic security (hides and wool) to the Asian and African countries.
2. SP and GP are highly infectious viral diseases that cause substantial economic losses by reducing productivity and increased susceptibility to other diseases and trade deficit by reducing free trade of animal and animal products from endemic areas to other places in another way [21,64,65].
3. The major reasons for the prevalence of SP and GP diseases include low production and coverage of the vaccines, poor quality management, transboundary movement of infected animals and animal products, and grazing in a common pasture with poor quality water [66,67].

## Conclusion

Infectious diseases are among the major factors which limit the production and productivity of small ruminants; SP and GP are prioritized in the list. SP and GP are often associated with high mortality and morbidity and significant economic loss to the producer. Hence, seroprevalence studies are essential to understand the spatial and temporal distribution of the diseases and tests that are very suitable for identifying, controlling, and eradicating the SP and GP diseases. From the systematic review and meta-analysis, it is concluded that regular vaccination with attenuated vaccines, education of farmers through extension activities, and effective transboundary movement restrictions leads to control and eradication of the diseases will be a reality in not in the Asians and African continent but all over the world. To the best of our knowledge, this study is the first to estimate the pooled prevalence of SP and GP in Asia and the African continent using systematic review and meta-analysis.

## Authors’ Contributions

KPS: Guided and supervised every step of the work. APB: Collected the data, conducted the analysis, and wrote the manuscript. CS: Analyzed the data. RRA: Drafted the manuscript. ES: Analyzed the data and drafted and edited the manuscript. VS: Extracted the data and edited the manuscript. SPK: Extraction and documentation of data. BRS: Reviewed and edited the manuscript. SSP: Extracted the data and drafted the manuscript. All authors read and approved the final manuscript.
